# Differential Patterns of Adherence to Opioid Therapy in Opioid Naïve and Opioid Existing Patients With Different Age Groups

**DOI:** 10.3389/fphar.2019.01286

**Published:** 2019-10-29

**Authors:** Che Suraya Zin, Nor Hidayah Taufek, Mazlila Meor Ahmad

**Affiliations:** ^1^Department of Pharmacy Practice, Kulliyyah of Pharmacy, International Islamic University Malaysia, Kuantan, Malaysia; ^2^Department of Anesthesiology and Intensive Care, Hospital Selayang, Batu Caves, Malaysia

**Keywords:** adherence, opioid therapy, opioid naïve, existing patients, proportion of days covered, patterns

## Abstract

Limited data are available on the adherence to opioid therapy and the influence of different patient groups on adherence. This study examined the patterns of adherence in opioid naïve and opioid existing patients with varying age and gender. This retrospective cohort study was conducted using the prescription databases in tertiary hospital settings in Malaysia from 2010 to 2016. Adult patients aged ≥18 years, receiving at least two opioid prescriptions, were included and stratified into the opioid naïve and existing patient groups. Adherence to opioid therapy was measured using the proportion of days covered (PDC), which was derived by dividing the total number of days covered with any opioids by the number of days in the follow-up period. Generalized linear modeling was used to assess factors associated with PDC. A total of 10,569 patients with 36,650 prescription episodes were included in the study. Of these, 91.7% (*n* = 9,696) were opioid naïve patients and 8.3% (*n* = 873) were opioid existing patients. The median PDC was 35.5% (interquartile range (IQR) 10.3–78.7%) and 26.8% (IQR 8.8–69.5%) for opioid naïve and opioid existing patients, respectively. A higher opioid daily dose (coefficient 0.010, confidence interval (CI) 0.009, 0.012 *p* < 0.0001) and increasing age (coefficient 0.002, CI 0.001, 0.003 *p* < 0.0001) were associated with higher levels of PDC, while lower PDC values were associated with male subjects (coefficient −0.0041, CI −0.072, −0.010 *p* = 0.009) and existing opioid patients (coefficient −0.134, CI −0.191, −0.077 *p* < 0.0001). The suboptimal adherence to opioid medications was commonly observed among patients with non-cancer pain, and the opioid existing patients were less adherent compared to opioid naïve patients. Increasing age and a higher daily opioid dose were factors associated with higher levels of adherence, while male and opioid existing patients were potential determinants for lower levels of adherence to opioid medications.

## Introduction

Medication adherence refers to the extent to which patients’ act of taking medication corresponds with the recommendations made by their health-care providers (Sabate, 2003). Non-adherence to medication has been associated with poor health outcomes (Sokol et al., 2005; Simpson et al., 2006), with more than 50% of non-adherence linked to long-term conditions (Vermeire et al., 2001; Dunbar-Jacob and Mortimer-Stephens, 2001). Chronic pain is one example of a long-term condition and is commonly treated with opioid analgesics. Monitoring adherence from the start of opioid therapy, in chronic pain, has reported a 50% lower incidence opioid abuse (Manchikanti et al., 2006) and may substantially reduce the costs and health issues associated with non-adherence to opioid therapy (McCarberg, 2011).

Adherence has commonly been studied in clinical conditions such as diabetes, asthma, and hypertension and is well characterized in different patient groups, including newly diagnosed and preexisting patients (Halpern et al., 2000). However, in patients with non-cancer pain treated with opioids, the adherence data are limited, and non-adherence to medication, in this population, is expected to occur to some extent (Ready et al., 1982; Berndt et al., 1993; Broekmans et al., 2009). Currently, it remains unclear whether the medication-taking behaviors in opioid naïve and opioid existing patients, in non-cancer pain, vary. Existing opioid patients have frequently been associated with long-term opioid use, with controversial reports available on the abuse, misuse, and opioid overdose-related deaths (Olsen, 2016), compared to the benefits of long-term use. Furthermore, the relationship between adherence and the detrimental effects associated with the long-term use of opioid warrants investigation. Previous studies in other clinical conditions have also reported that elderly patients were commonly associated with an increased risk of poor adherence due to cognitive and function impairment (Helling et al., 1987; Sabate, 2003). Moreover, data on adherence levels in different age groups among patients with non-cancer pain are lacking.

For the adherence measure, a cutoff point of 80% has been commonly used in previous studies (Broekmans et al., 2009; Timmerman et al., 2016). However, this may not reflect the real medication-taking behavior, and the patient’s adherence behavior may likely be misclassified and misinterpreted. A wide range exists between 0% and 80%, and it is inaccurate to assume that these ranges reflect the same medication-taking behavior (Baker et al., 2017). In contrast, the continuous measure of adherence provides rich information on the medication-taking behavior lacking in most previous studies.

The present study was conducted in patients with non-cancer pain in an attempt to describe the patterns of adherence behaviors in opioid naïve and opioid existing patients of different ages and gender. To close the gap of limited information available when the adherence measure uses a cutoff point of 80%, this study used a continuous measure of adherence to ensure a more accurate reflection of patients’ medicine-taking behaviors. A better understanding of the differential patterns of adherence in opioid medications, among different patient groups, is important in identifying patients at risk of poor adherence, necessitating appropriate monitoring during therapy.

## Methods

This study used de-identified data and reported the results in an aggregated manner. There was no direct patient involvement in this study, thus waiving the requirement for informed consent. Ethical approval was obtained from the Medical Research Ethical Committee, Ministry of Health, Malaysia (NMRR-16-2135-33068).

### Study Design and Setting

This was a retrospective cohort study conducted at two tertiary outpatient hospitals in Malaysia. These hospitals had ∼800‒1,000 inpatient beds per hospital and provided various facilities including pain-relief services, renal services, surgery, and anesthesiology. The prescription database from 1^st^ January 2010 to 31^st^ December 2016 was accessed for details on prescription data such as drug names, strengths, quantities, frequencies, duration, issuing departments, and prescription dates. This study included all available opioids at these hospitals (buprenorphine, morphine, oxycodone, fentanyl, dihydrocodeine, and tramadol).

The patients’ age and sex were extracted from the prescription database. The patients’ age was calculated based on the date of first opioid prescription entered in the database, for example, if a patient has two opioid prescriptions during the study period (the first prescription was on 1/1/2011 and the second prescription was on 15/2/2012) and patient’s date of birth was on 4/10/1970. The age was then calculated by subtracting the date of the first prescription by the date of birth (2011–1970 = 41 years old). Only the year was included in the calculation.

Patients aged ≥18 years with at least two opioid prescriptions were included. They were stratified into five age groups (18 to 40, 41 to 50, 51 to 60, 61 to 80, and ≥81 years old). These patients were followed up from their first opioid prescription (index date) until the discontinuation of opioid treatment, or until the end of the study period on 31^st^ December 2016, or death by any cause, whichever occurred first. Patients with only one opioid prescription were excluded from the study (Halpern et al., 2000).

Patients were categorized into the opioid naïve group if they had not received any opioid prescription in the year 2010, and the opioid existing group if they had received an opioid prescription in a previous year (2010) prior to the study commencement (1^st^ January 2011). For prescriptions of tramadol, oxycodone, fentanyl, dihydrocodeine, and buprenorphine that were written on an as needed basis (*prn*), the imputation of frequency was conducted based on last observation carry forward (Bohnert et al., 2016). However, prescriptions with morphine written on an as needed basis (*prn*) were excluded from the analysis as these were most commonly used for cancer-associated pain. Opioid prescriptions from the palliative care units were also excluded to ensure only patients with non-cancer pain were included in this study. Prescriptions for methadone, which were used exclusively for opioid addiction, were also excluded.

### Measurement of Adherence

This study used proportion of days covered (PDC) to measure the adherence. PDC was derived by dividing the total number of days covered with any opioid drug by the number of days in the follow-up period (Raebel et al., 2013).

$$\text{PDC}=\frac{\text{Number of days in period}"\mathrm{covered}"}{\text{Number of days in period}}\times 100\%$$

The total number of days covered with opioid prescriptions was derived by summing up all days of supply of opioid prescriptions for each patient for a particular follow-up period. The days of supply of each opioid prescription were calculated by dividing the quantity of opioids supplied by the number of daily doses (frequency). In case patients were receiving multiple opioid prescriptions on the same day, the prescription with the largest number of days’ supply was included. If the prescriptions overlapped (the second prescription was issued before the duration of the first prescription came to an end), the overlapping period was subtracted.

A continuous measure of PDC, for both opioid naïve and opioid existing patients, was recorded. As previous studies that used a continuous measure of adherence were unavailable, this study also recorded a dichotomous measure, PDC < 80% (refer to non-adherence) and ≥80% (adherence) for comparison purposes. The terms “non-adherence” and “suboptimal adherence” were used interchangeably in this study. Non-adherence is commonly used in studies using dichotomous measures of adherence, while sub-optimal is used for continuous measure (0–80%).

### Calculation of Opioid Dose Per Day

The oral morphine milligram equivalents (MMEs) of each opioid prescription were calculated by multiplying the opioid dose by the conversion factor in accordance with the Centers for Disease Control and Prevention (CDC) (CDC Compilation of Opioid Analgesic Formulations With Morphine Milligram Equivalent Conversion Factors, 2013). The opioid dose for each prescription was summed up across all prescriptions for each patient, for a particular follow-up period, to derive the total opioid dose in MMEs. This total opioid dose was then divided by the total days of supply for each patient, for a particular follow-up period, to derive the opioid dose per day for each patient in MMEs. For patients with multiple opioid prescriptions on the same day, a combined daily dose in MMEs was calculated by merging all the doses.

### Data Analysis

Patient characteristics were described using descriptive statistics. Continuous variables were presented as a mean with SD for normally distributed data and median with interquartile range (IQR) for non-normally distributed data. Categorical variables were presented as proportion. *Gamma* distribution was used to model the skewed distribution of continuous data of PDC, and as PDC cannot be in negative counts, this study then employed a log link function in the model. As such, to measure the influence of covariates on adherence to opioid therapy, this study used a generalized linear model (GLM) with gamma family and log-link function, with PDC for opioid therapy as the dependent variable. Patients’ age, sex, and opioid dose per day were included as covariates (independent variables), as they may influence patients’ adherence. The type of opioid issued on the index date was also included as one of the covariates. Regression coefficients and 95% confidence intervals (CIs) were used to present the results, which were considered statistically significant for a *p*-value <0.05. All analyses were performed using the Stata v15.1 software (StataCorp LLC, 2015) (“StataCorp. Stata: Release 15. College Station, TX: StataCorp LLC; 2015.,” n.d.).

## Results

### Baseline Characteristics

**Figure 1:**
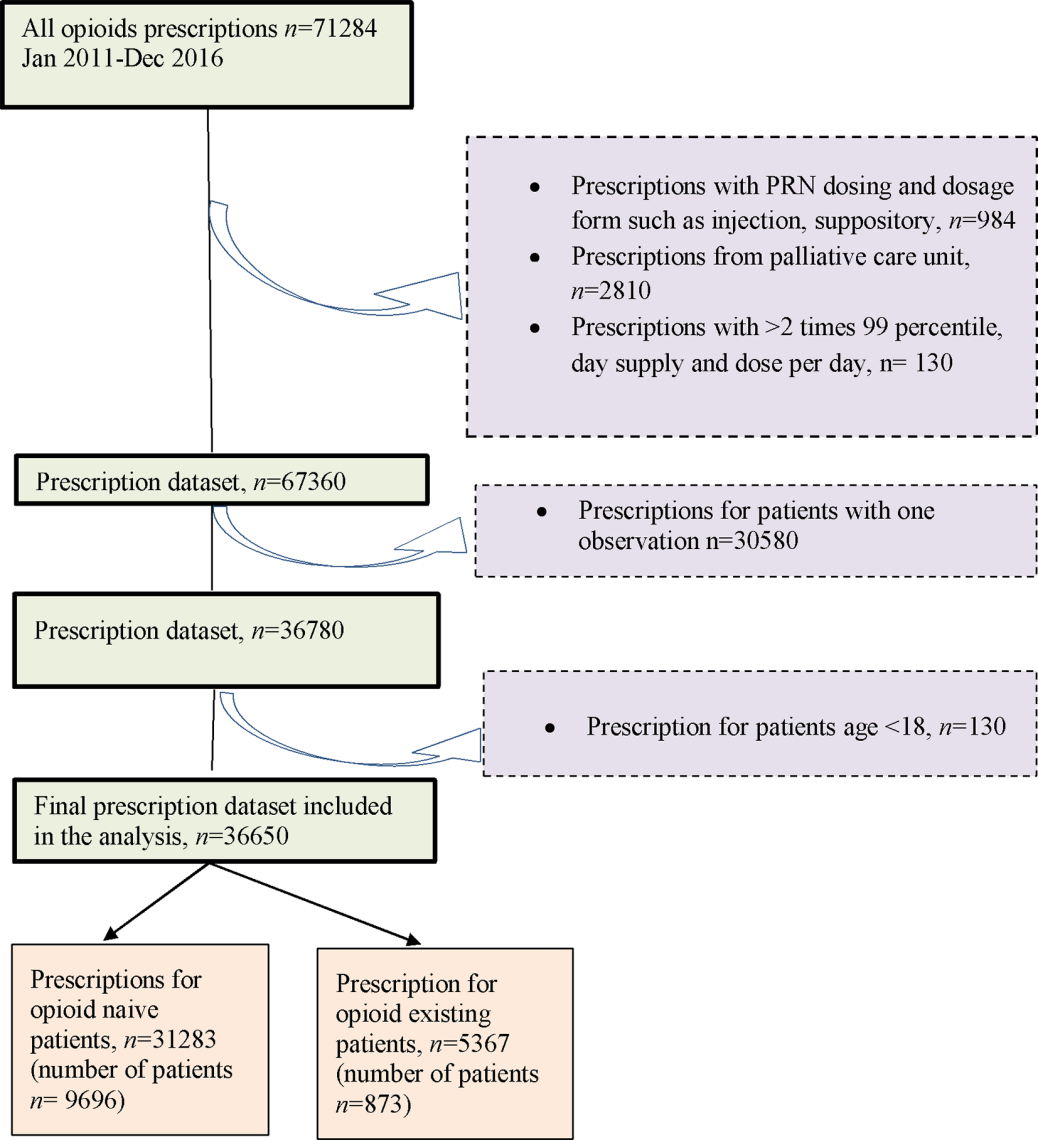
Cohort flow chart.

Overall, this study included 10,569 opioid patients with 36,650 prescription episodes including six opioid drugs identified during the follow-up period. Of the total number of patients, 91.7% (*n* = 9,696) were opioid naïve patients and 8.3% (*n* = 873) were opioid existing patients (**Figure 1**) with 50.7% and 56.2% females in each of the two groups, respectively.

**Table 1** demonstrates that the mean (SD) age of patients, at baseline, was 55.4 (16.1) years for the opioid naïve patients and 61.1 (14.8) years for the opioid existing patients. In the opioid naïve group, patients aged between 61 and 80 years were predominant (36.3%), followed by 51 to 60 (23.9%), 18 to 40 (20.3%), 41 to 50 (15.0%), and ≥81 years (4.4%). In the opioid existing group, the largest group of patients was aged between 61 and 80 years (42.8%), followed by 51 to 60 (25.5%), 41 to 50 (11.9%), 18 to 40 (10.1%), and ≥81 years (9.6%). The median follow-up durations for opioid naïve and opioid existing patients were 0.7 (IQR 0.3–1.9) years and 2.5 (IQR 0.9–4.2) years, respectively.

**Table 1:** Patient demographics.

Type of patients	Naïve patients		Existing patients		Total patients	
	*n*	%	*n*	%	*n*	%
Number of patients	9,696	91.7	873	8.3	10,569	100
*Gender*						
Male	4,779	49.3	382	43.8	5,161	48.8
Female	4,917	50.7	491	56.3	5,408	51.2
*Age*						
Mean	55.4		61.1		55.9	
Median	57		62		57	
Mode	55		59		55	
Range	18–105		18–96		18–105	
SD	16.1		14.8		16.1	
*Age group*						
18 to 40 years old	1,970	20.3	88	10.1	2,058	19.5
41 to 50 years old	1,456	15.0	104	11.9	1,560	14.8
51 to 60 years old	2,319	23.9	223	25.5	2,542	24.1
61 to 80 years old	3,521	36.3	374	42.8	3,895	36.9
≥81 years old	430	4.4	84	9.6	514	4.9
*Type of opioids*						
Buprenorphine	163	0.52	34	0.63	197	0.5
Dihydrocodeine	175	0.56	11	0.2	186	0.5
Fentanyl	202	0.65	6	0.11	208	0.6
Morphine	636	2.0	284	5.3	920	2.5
Oxycodone	946	3.0	13	0.2	959	2.6
Tramadol	29,161	93.2	5,019	93.5	34,180	93.3
Total prescriptions	31,283	100.0	5,367	100.0	36,650	100.0
Median follow-up time (year, IQR)	0.7	0.3–1.9	2.8	0.9–4.2	0.8	0.3–2.1
Median dose/day (mg, IQR)	30.0	30.0–30.6	30.0	30.0–32.4	30.0	30.0–30.8

In the 36,650 prescribing episodes, the most commonly prescribed drug, in the opioid naïve patients, was tramadol (93.2%), followed by oxycodone (3.0%), morphine (2.0%), fentanyl (0.7%), dihydrocodeine (0.6%), and buprenorphine (0.5%). In the opioid existing patients, tramadol was the most frequently prescribed (93.5%), followed by morphine (5.29%), buprenorphine (0.63%), oxycodone (0.24%), dihydrocodeine (0.2%), and fentanyl (0.11%).

### Adherence Measure

**Figure 2:**
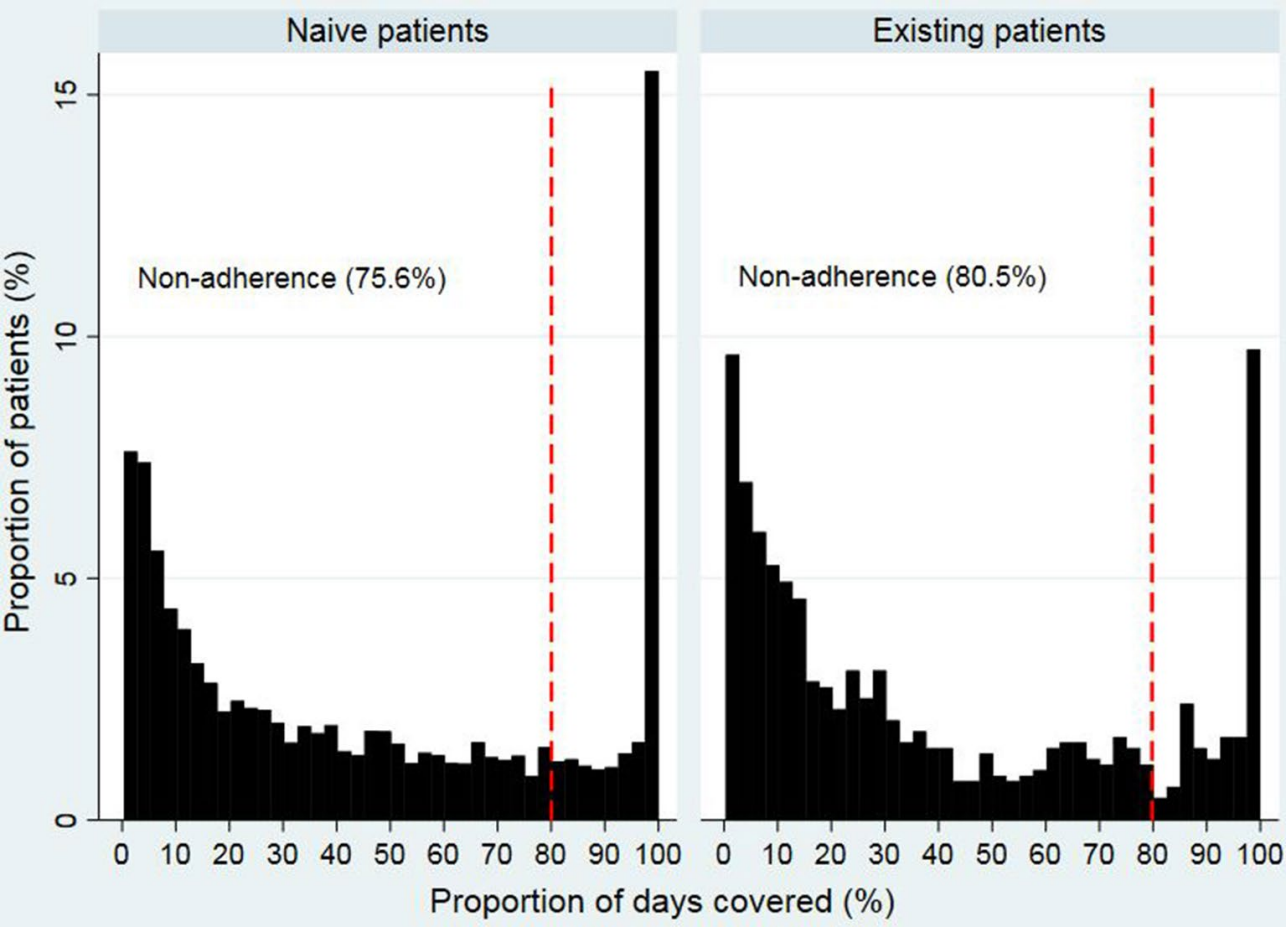
Proportion of days covered in opioid naïve and opioid existing patients.

**Figure 3:**
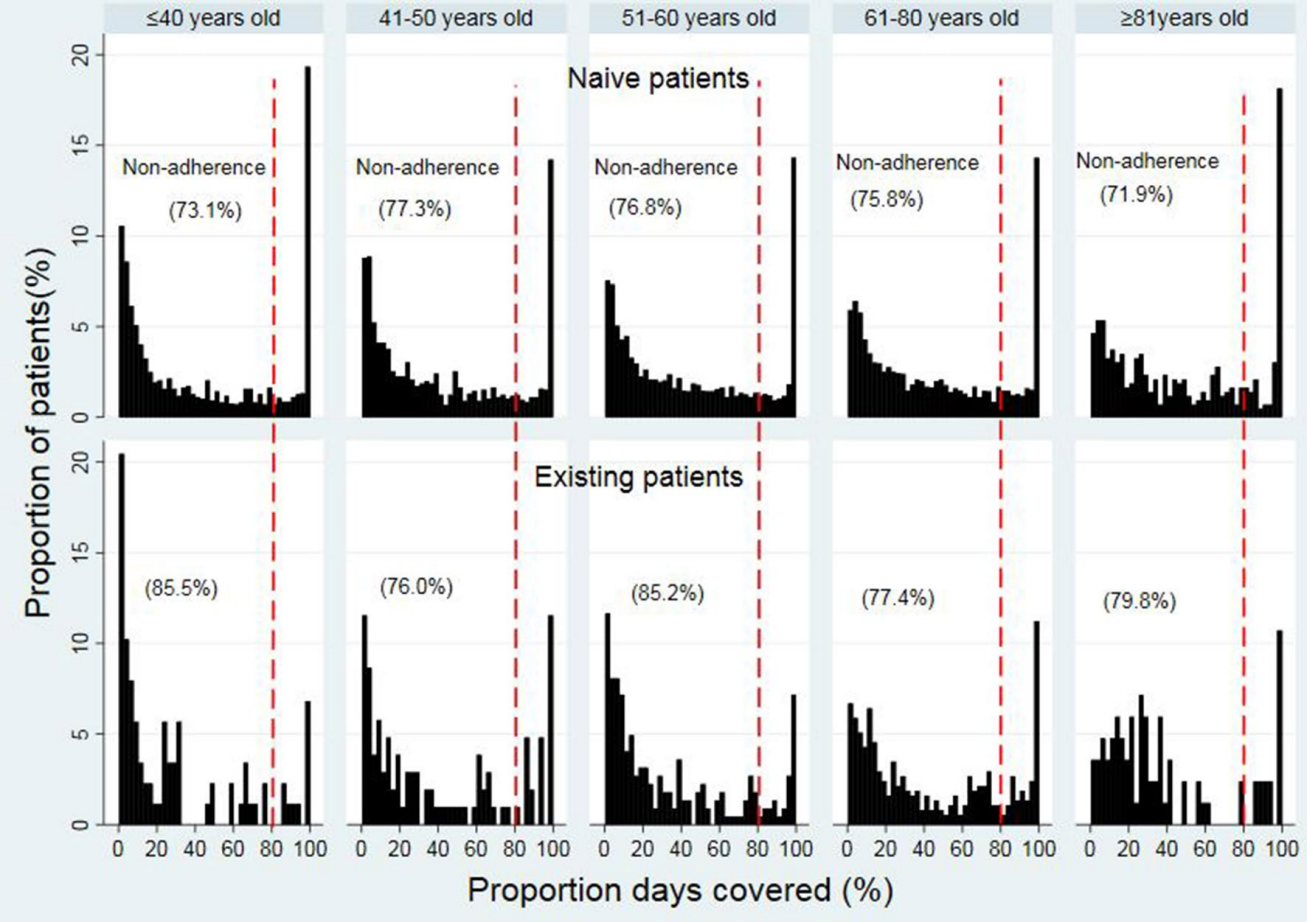
Proportion days covered in opioid naïve and existing patients in different age groups.

**Figures 2** and **3** show that the distribution of PDC for both opioid naïve and opioid existing patients, as well as the PDC for patients of different ages in both groups, was not normally distributed. The median PDC was 35.5% (IQR 10.3%–78.7%) for opioid naïve and 26.8% (IQR 8.8%–69.5%) for opioid existing patients. Mean PDC for opioid naïve and opioid existing patients was 44.2% and 39.3%, respectively. The dichotomous adherence measure showed that 75.6% of opioid naïve and 80.5% of opioid existing patients demonstrated non-adherence (PDC < 80%) to opioid medications.

The median PDC values among different age groups ranged from 31.4% to 43.8% in opioid naïve patients within different age groups (31.4% (IQR 7.7%–84.5%), 32.4% (IQR 8.9%–74.8), 34.6% (IQR 10.7%–76.0%), 38.1% (IQR 12.2%–78.5%), and 43.8% (IQR 15.2%–85.2%) for 18 to 40, 41 to 50, 51 to 60, 61 to 80, and ≥81 years old, respectively) (**Figure 3**). For opioid existing patients, the median PDC values ranged from 15.5% to 33.2%. Details of the PDC (15.5% (IQR 3.7%–48.8%), 28.1% (8.1%–75.5%), 20.7% (IQR 7.0%–59.5%), 33.2% (IQR 11.6%–74.3%), and 28.3% (15.1%, 57.5%) for 18 to 40, 41 to 50, 51 to 60, 61 to 80, and ≥81 years old, respectively).

The mean PDC for patients in different age groups was (43.7% vs. 29.5%) for 18 to 40, (42.2% vs. 41.1%) for 41 to 50, (43.7% vs. 34.4%) for the 51 to 60, and (45.2% vs. 43.9%) for 61 to 80, and (49.1% vs. 39.6%) for ≥81 years, accordingly, in opioid naïve and opioid existing patients.

The trend of non-adherence (using the dichotomous measure of adherence) (< 80% PDC) was improving in the older patients in opioid naïve while it was fluctuating in the opioid existing patients across the age groups. In the opioid naïve group, non-adherence (< 80% PDC) to opioid therapy was the highest in patients in ages of 41 to 50 years (77.3%), followed by 51 to 60 (76.8%), 61 to 80 (75.8%), 18 to 40 (73.1%), and ≥81 years (71.9%) (**Figure 3**). In the existing opioid patient group, the non-adherence (< 80% PDC) was the highest in patients in the ages of 18 to 40 years (85.5%), followed by 51 to 60 (85.2%), ≥81 (79.8%), 61 to 80 (77.4%), and 41 to 50 years (76.0%).

When patients were stratified into ≤60 years (younger group) and >60 years old (older group), the overall mean of non-adherence (< 80% PDC) was higher in the younger patients (≤60 years) than in the older patients (> 60 years) in both study groups. Details of the overall non-adherence were (75.7% vs. 73.9%) in the opioid naïve and (82.2% vs. 78.6%) in the opioid existing groups, respectively, for patients’ age ≤60 and >60 years.

The GLM analysis demonstrated that higher daily opioid doses (coefficient 0.010, 95% CI 0.009, 0.012 *p* < 0.0001) and increasing age (coefficient 0.002, 95% CI 0.001, 0.003 *p* < 0.0001) were associated with higher levels of PDC (**Table 2**). On the other hand, male (coefficient −0.0041, CI −0.072, −0.010 *p* = 0.009) and existing opioid patients (coefficient −0.134, CI −0.191, −0.077 *p* < 0.0001) were associated with lower levels of PDC. Lower PDCs were also associated with patients who were initiated with opioids such as tramadol, oxycodone, fentanyl, dihydrocodeine, and morphine during the study period. However, this association was not significant (*p* > 0.05).

**Table 2:** Results from the generalized linear model regression of the factors influenced adherence.

	Coefficients	Lower 95% CI	Upper 95% CI	*p* value
Patient groups				
Opioid naïve patients	1			
Opioid existing patients	−0.134	−0.191	−0.077	*p* = 0.0001
Age	0.002	0.001	0.003	*p* = 0.0001
Sex				
Female	1			
Male	−0.041	−0.072	−0.010	0.009
Opioid dose per day	0.010	0.009	0.012	*p* = 0.0001
Individual opioid				
Buprenorphine	1			
Dihydrocodeine	−0.044	−0.802	0.714	0.910
Fentanyl	−0.192	−0.906	0.522	0.598
Morphine	−0.129	−0.795	0.536	0.703
Oxycodone	−0.174	−0.843	0.496	0.611
Tramadol	−0.512	−1.164	0.139	0.123

### Opioid Dose Per Day

The median opioid dose per day was similar in both opioid naïve (30.0 mg/day, IQR 30.0–30.6 mg/day) and existing opioid patients (30.0 mg/day, IQR 30.0–32.4 mg/day) (**Table 1**). The mean (SD) of the dose per day was similar, 33.5 (15.6) mg/day in naïve patients and 34.3 (19.7) mg/day in existing patients.

## Discussion

This study reported that the majority of patients with non-cancer pain demonstrated suboptimal adherence to opioid medications. The median PDC was 35.5% in opioid naïve and 26.8% in opioid existing patients, indicating that the opioid existing patient group was less adherent to opioid therapy compared to the opioid naïve patients.

A direct comparison with other studies was difficult as the current study analyzed adherence using a continuous variable, while previous studies mostly reported data on adherence as dichotomous variables (adherence vs. non-adherence) (Broekmans et al., 2009; Timmerman et al., 2016). The continuous variable used in the current study provided an overview on the differential patterns of medication-taking behavior, while the dichotomous variable is limited in providing such information on adherence and might oversimplify the measure (MacCallum et al., 2002). The dichotomous variable is unable to explain complex behavioral patterns in medication-taking behaviors and may underestimate non-adherence due to the nature of its assessment (e.g., self-report and interview) being associated with recall bias or social desirability (Broekmans et al., 2010).

Although previous studies reporting a continuous variable in non-cancer pain were unavailable, using the dichotomous adherence measure, the current study demonstrated that 75.6% of opioid naïve and 80.5% of opioid existing patients were associated with non-adherence (PDC < 80%) to opioid therapy. These findings support the observations reported in previous studies which highlighted that non-adherence to opioid medications was common in patients with non-cancer pain (Broekmans et al., 2009; Timmerman et al., 2016). It was indicated that the proportion of non-adherence to analgesic medications in chronic non-cancer pain ranged from 8% to 62% (Timmerman et al., 2016). However, these studies reported on the adherence to all analgesic medications, and not exclusively to opioids, which may explain the slightly lower rate of non-adherence compared to the present study. Moreover, the subjective methods such as self-report and interviews used in previous studies tend to underestimate non-adherence (LaFleur and Oderda, 2004). The high rate of non-adherence in patients with chronic non-cancer pain may be explained by the non-life-threatening nature of these conditions, which leads the patients to perceive that non-adherence to medication-taking has no immediate effects on the disease outcomes (Broekmans et al., 2009).

With regard to the adherence between different age groups, the present study found that the non-adherence (using dichotomous measure of adherence) to opioid therapy (< 80% PDC) was higher in younger patients than in older patients. The age groups that were associated with the highest non-adherence to opioid medications were the 41 to 50-year group in opioid naïve patients and the 18 to 40-year group in opioid existing patients. In the GLM, increasing age (coefficient 0.002, 95% CI 0.001, 0.003 *p* < 0.0001) was associated with higher adherence levels. This finding strongly suggests that younger patients were less likely to adhere to opioid therapy compared to elderly patients, which is congruent with previous studies that have reported a positive association between age and analgesic adherence (Dunbar-Jacob and Mortimer-Stephens 2001; Broekmans et al., 2010; Grattan et al., 2012). Conversely, few studies have also reported the absence of an association between age and medication adherence (Susan Broekmans et al., 2010; Markotic and Obrdalj 2013), and also a negative association (Timmerman et al., 2014). The differences in these findings are probably due to different methods used in these studies as described above. In other disease conditions such as diabetes and hypertension, the non-adherence was also higher in young patients (Briesacher et al., 2008) and consistent with the present study.

The low adherence to opioid therapy in young patients may require attention, as the risk for abuse of prescribed opioid medications is reportedly the highest among young patients (Banta-green et al., 2009), and poor adherence is one of the likely factors that contribute to this risk. In case of older patients with age >60 years, the overall adherence to opioids is still suboptimal (median and the mean PDC < 50%) despite being higher than in younger patients. This could be attributed to the cognitive and functional impairments which are commonly associated with older age, thus increasing the risk of suboptimal adherence (Markotic and Obrdalj, 2013).

The present study also found that an increasing opioid dose per day (coefficient 0.010, CI 0.009, 0.012 *p* < 0.0001) was associated with higher levels of adherence. This may reveal that patients are more likely to adhere to their medicine when the severity of pain is high, or when higher pain intensities are experienced, as higher doses are commonly prescribed in cases experiencing severe pain. Previous studies have reported similar observations, with patients in poorer health or experiencing a higher pain intensity reportedly more likely to be adherent to analgesic medications (DiMatteo et al., 2007; Broekmans et al., 2010; Markotic and Obrdalj, 2013). However, the current study was not able to evaluate pain intensity due to the retrospective nature of the study and the use of a prescription database. Overall, this study also showed that the majority of opioid naïve and opioid existing patients were using a low dose of opioids <50 mg/day, and this corresponded to a study previously conducted on the dose and duration of opioids in cancer and non-cancer pain in a tertiary hospital setting in Malaysia (Zin et al., 2017).

This study also demonstrated that men were associated with less adherent behavior to opioid therapy (male, −0.0041, CI −0.072, −0.010 *p* = 0.009) compared to women, indicating the need for close monitoring of opioid therapy in men. This finding was consistent with a previous study in patients with chronic non-cancer pain that reported men (39.6%) were less adherent than women to therapy (60.4%) (Broekmans et al., 2010). The differential adherence behavior to opioid therapy in men compared to women may explain the higher risk of men to escalate to higher doses of opioid therapy and death from opioid-related causes (Kaplovitch et al., 2015; Zin et al., 2019) compared to women, despite men being reportedly less likely to use prescription opioids compared to women (Campbell et al., 2010; Serdarevic et al., 2017)

The current study also observed that opioid existing patients were associated with lower levels of adherence (coefficient −0.134, CI −0.191, −0.077 *p* < 0.0001). The median follow-up time for the existing patients was 2.47 years, reflecting the long-term use of opioids. The suboptimal adherence in the opioid existing patient group may partly explain the association with high risks of misuse, abuse, and overdose-related death (Bohnert et al., 2014; Olsen 2016).

The strength of the current study was the assessment of adherence to opioid therapy in both opioid naïve and opioid existing patients. The objective to measure the adherence using proportion days covered is preferable to subjective measures such as self-report and interview as it estimates the adherence in a more conservative way (Nau, 2012). The continuous variable provides more information on adherence behavior compared to the dichotomous binary variable of a cutoff point of 80%, which is an arbitrary and non-empirical method (Kronish et al., 2011). However, this study has a number of limitations. The prescription data and retrospective nature of this study design, with the unavailability of relevant information such as pain intensity and comorbidities, may confound the associations outlined in this study. The use of PDC as a proxy for adherence may overestimate the adherence to medication, as it was indirectly measured using the prescribing data. Although there are many methods of measuring adherence using secondary databases, the adherence values of the various methods were comparable, and no issues in measuring adherence were encountered (Hess et al., 2006). The findings from an outpatient hospital setting might not be easily generalized to other settings, including private, primary care, or inpatient settings. By using the prescribing data, we assumed that patients would adhere to treatment; however, it cannot be ensured that the patients actually take the medication. This is a common problem affecting most investigations and is not unique to this study (Caetano et al., 2006).

## Conclusion

The overall adherence to opioid medications was suboptimal among patients with non-cancer pain. Opioid existing patients were less adherent to opioid therapy compared to the opioid naïve patients. Increasing age and a larger daily opioid dose was associated with higher levels of adherence, while male and existing opioid patients were the potential determinants for lower levels of adherence to opioid therapy. Patients at risk of poor adherence to opioid therapy could be identified using the available information on adherence patterns, and appropriate monitoring measures could be instituted. This information may guide and determine the need for patient education to improve adherence level. Health-care providers need to clearly communicate the goals of pain treatment and the issues with opioid therapy towards improving patients’ adherence.

## Data Availability Statement

Requests to access the datasets should be directed to Medical Research Ethical Committee, Ministry of Health Malaysia, as ethical approval is required to access the data. The data is restricted to maintain the privacy and confidentiality of patients’ health records.

## Ethics Statement 

This study obtained the ethical approval from the Medical Research Ethical Committee, Ministry of Health, Malaysia (NMRR-16-2135-33068). Written informed consent for participation was not required for this study in accordance with the national legislation and the institutional requirements.

## Author Contributions

CZ initiated and developed the research questions and study design, conducted data management and analysis, and led drafted the manuscript. The rest of the authors (NT and MA) contributed to the data acquisition and interpretation of the data, critically revised the manuscript, and approved the final version submitted for publication.

## Funding

CZ was Supported by a Research Grant From the Ministry of Education Malaysia (Fundamental Research Grant Scheme, FRGS 19-010-0618). The funder had no role in study design, data collection and analysis, decision to publish, or preparation of the manuscript.

## Conflict of Interest

The authors declare that the research was conducted in the absence of any commercial or financial relationships that could be construed as a potential conflict of interest.
